# Gestational Health Outcomes Among Pregnant Women in the United States by Level of Dairy Consumption and Quality of Diet, NHANES 2003–2016

**DOI:** 10.1007/s10995-022-03469-4

**Published:** 2022-08-08

**Authors:** Benjamin J. K. Davis, Xiaoyu Bi, Kelly A. Higgins, Carolyn G. Scrafford

**Affiliations:** Center for Chemical Regulation and Food Safety, Exponent, Inc, 1150 Connecticut Avenue, NW, Suite 1100, Washington, DC 20036 USA

**Keywords:** Pregnancy, Diet quality, Dairy consumption, Gestational diabetes mellitus, Gestational weight gain

## Abstract

**Objectives:**

Diet is an important factor in gestational health. Many pregnant women have suboptimal diets and dairy foods are an excellent source of key nutrients. The aim of this work was to investigate the relationships between dairy consumption (cup equivalents/day) or diet quality assessed using the Healthy Eating Index-2015 and gestational diabetes mellitus (GDM) or gestational weight gain (GWG) among pregnant women in the United States (US).

**Methods:**

Study populations were subsets of pregnant, non-lactating women (20–44 years) in the National Health and Nutrition Examination Surveys 2003–2016, which was approved by the National Center for Health Statistics Research Ethics Review Board. GDM and GWG were classified according to national guidelines. General characteristics were compared across categories of dietary variables. Adjusted regression models estimated associations between diet and GDM and GWG.

**Results:**

No statistically significant linear associations between dairy consumption or diet quality and GDM or GWG were observed.

**Conclusions for Practice:**

Future research should aim to address the limitations of the current cross-sectional analyses and further elucidate the underlying relationships between diet and gestational health.

## Significance

*What is already known on this subject?* Diet quality and dairy consumption may be protective of gestational health, in particular gestational diabetes and gestational weight gain.

*What this study adds?* Evidence is limited and/or inconsistent regarding the relationship between either dairy products or diet quality and gestational health outcomes. In the current study, no significant linear associations were identified between diet quality, dairy consumption, and gestational health outcomes when using a large survey representative of the US population. These findings highlight the need to conduct studies that are specifically designed to evaluate the relationship between diet and gestational health.

## Introduction

A balanced and nutrient-dense diet is an important factor in gestational health, including reducing the risk for gestational diabetes mellitus (GDM) and maintaining appropriate gestational weight gain (GWG) during pregnancy. However, a large proportion of pregnant women have nutrient intakes below recommended levels (Bailey et al., 2019). GDM and excessive or inadequate GWG are public health concerns, with prevalence as high as 10% and 69% in the United States (US), respectively (Centers for Disease Control & Prevention, 2019; QuickStats:, 2016). GDM and inadequate or excessive GWG are associated with adverse health effects in both women during pregnancy and offspring at birth and later in life (Hedderson et al., 2010; Heude et al., 2012; Rasmussen & Yaktine, 2009).

The Dietary Guidelines for Americans (DGA) provide recommendations to help the US population meet nutrient needs (USDA, 2015). Dairy foods, a component of the DGA recommendations, are a rich source of many nutrients, however, the majority of pregnant women in the US report consumption well below the recommended levels (DGAC, 2020; USDA, 2015). Evidence is limited and/or inconsistent regarding the relationship between either dairy products or diet quality and gestational health outcomes. Protective effects of low‐fat dairy intake during pregnancy, evaluated both as an individual component and part of a dietary pattern, on the risk of GDM have been observed in cohorts of pregnant women outside the US (Osorio-Yáñez et al., 2017; Sartorelli et al., 2019; Zareei et al., 2018). An increased odds of GDM with diets low in milk and cheese and high in nuts, seeds, fat, and soybean has been reported among US pregnant woman (Shin et al., 2015). As part of the Pregnancy and Birth to 24 Months Project, a systematic review of dietary patterns and risk of GDM concluded the evidence was insufficient to estimate an association during pregnancy (Raghavan et al., 2019). The relationship between diet and GWG was reviewed by the 2020 US Dietary Guidelines Advisory Committee (DGAC) which noted inconsistent associations (DGAC/NESRT, 2020). When dairy was evaluated as part of a dietary pattern, consumption of low-fat dairy products or total dairy in moderation was observed to be beneficial for adequate GWG, while others noted associations with excess GWG (DGAC/NESRT, 2020). Recent studies on GWG and dairy that were not considered in the DGAC report remain inconsistent or limited to small observational cohorts (Hirko et al., 2020; Lai et al., 2019; Mukhopadhyay et al., 2018). When considering overall diet quality, studies measuring adherence to the dietary patterns recommended by the DGA through the Healthy Eating Index (HEI) reported null associations with GWG (Schlaff et al., 2020; Shin et al., 2014). However, these studies were based on older survey data or were conducted in a limited population.

Diet quality and dairy consumption may play a beneficial role in gestational health. To our knowledge, an evaluation of the relationships between dairy intake or diet quality, as measured by the HEI-2015, and GDM or GWG in a nationally representative sample has not been conducted. The primary objective of this study is to quantify these associations among pregnant women in the US.

## Methods

### Study Populations

This cross-sectional study was conducted with data collected in the combined 2003–2016 National Health and Nutrition Examination Survey (NHANES) and the dietary recall component known as what we eat in America (WWEIA) (National Center for Health Statistics, 2020). The NHANES is designed to provide nationally representative nutrition and health data and prevalence estimates for nutrition and health status measures in the US (Ahluwalia et al., 2016). The NHANES assessment includes an in-person household interview, a health examination in a mobile examination center (MEC), and a telephone follow-up interview 3–10 days after the MEC examination. Approval for the NHANES data collection was provided by the National Center for Health Statistics (NCHS) Research Ethics Review Board and the survey was conducted in accordance with ethical standards in the 1964 Helsinki Declaration and later amendments. All participants gave informed consent prior to inclusion in the survey.

The source population was limited to pregnant, non-lactating women (20–44 years) who were classified based on a positive urine test and who provided a reliable dietary recall on Day 1 by meeting the minimum criteria as determined by NCHS (n = 791). In the GDM analysis, after exclusions due to lack of MEC morning exam data (n = 419), not fasting (n = 43), and history of type I or II diabetes (n = 8), the final sample was 321. In the GWG analysis, after exclusion of pregnant women in the NHANES 2013–2016 cycles due to the lack of data on gestational age (n = 200) and women missing anthropometric measures (n = 10), the final sample size was 581. Therefore, this sample included only participants reporting gestational age in months and self-reported weight before pregnancy.

### Definition of Gestational Health Outcomes

GDM was diagnosed based on fasting plasma glucose (FPG) threshold of 5.1 mmol/L from the American Diabetes Association’s one-step oral-glucose tolerance test (OGTT) strategy (American Diabetes Association, 2020). GWG was calculated as the difference between each woman’s measured weight at the time of examination and self-reported pre-pregnancy weight. Each woman’s GWG was classified according to the US Institute of Medicine’s criteria as “adequate”, “excessive”, or “inadequate” (Rasmussen & Yaktine, 2009).

### Dietary Assessment

#### Dairy Consumption

Women were categorized based on total servings of dairy per day (as cup equivalents/day; cup-eq/d) reported in their Day 1 dietary recall using the following cut-points: < 1, 1 to < 2.5, and ≥ 2.5 cup-eq/d. The US Department of Agriculture’s (USDA’s) MyPyramid Equivalent Database and Food Pattern Equivalents Database were used to determine total servings of dairy intake for 2003–2004 and 2005–2016, respectively (Bowman et al., 2019). Differences in the databases between food component categorizations were remapped for consistency. The dairy component represents milk, yogurt, cheese, and miscellaneous dairy.

#### Diet Quality

Diet quality was assessed with the HEI-2015, designed to roughly measure adherence to the 2015–2020 DGA among US adults (Krebs-Smith et al., 2018). HEI-2015 includes 13 components consisting of adequate intake of nutrient dense food components, such as fruits and vegetables, and limiting intake of food components to consume in moderation, such as solid fats and added sugars. The maximum sum of the components is 100 points, indicating perfect adherence; the score for the average adult is much lower (~ 60 points; NCHS, 2020). Each individual’s HEI-2015 total score was calculated in all survey cycles using the simple HEI scoring algorithm method from the National Cancer Institute developed SAS macros (National Cancer Institute, 2020). The reported consumption of each HEI component was based on the same linkages to survey-specific USDA databases as described above. Differences in the databases between food component categorizations across years were remapped for consistency. The HEI-2015 total score was used to categorize each pregnant woman in the sample into tertiles of diet quality.

### Statistical Analysis

Analyses for GDM and GWG outcomes were conducted independently. Characteristics of the NHANES participants were summarized for each study population sample. Logistic regression models were used to estimate odds of diagnosis of GDM among pregnant women in each of the dairy intake and HEI-2015 categories. Multinomial logistic regression models with adequate GWG as the reference category were used to estimate prevalence ratios of inadequate and excessive GWG among pregnant women in each of the dairy intake and HEI-2015 categories. Model 1 adjusted for age and total energy intake (dairy intake only). Model 2 further adjusted for potential confounders including parity, marital status, race/ethnicity, education status, household poverty income ratio, pre-pregnancy body mass index (BMI), smoking status, physical activity, dietary supplement use, and history/diagnosis of diabetes mellitus (GWG only). Model 3 (dairy intake only) further adjusted for dietary intake of fat, fiber, protein, and added sugars. All models were analyzed using participants with complete records of included variables.

All statistical analyses were performed using STATA (version 11.2, 2011, StataCorp LP, College Station, TX, USA) and conducted using appropriate statistical weights and adjusted for design effect so that all results are nationally representative of civilian, non-institutionalized pregnant women in the US. Specifically, the GWG analyses were performed using the day 1 dietary recall weights, while the GDM analyses were performed using the fasting subsample MEC weights. An α of 0.05 was set for all regression analyses.

## Results

The characteristics of the study population are presented in Table 1. The average age of women in both the GDM and GWG samples was approximately 29 years. Pregnant women in the study samples were mostly white, married, multiparous, never-smokers, and had at least some college education. Average dairy consumption was slightly higher than the national average at approximately 2 cup-eq/d (DGAC, 2020), with an average HEI-2015 of 50, which is slightly lower than the national average (NCHS, 2020).

**Table 1:** Characteristics of the study populations

	Gestational weight gain study population		Gestational diabetes mellitus study population	
	N	Value	N	Value
Total dairy consumption (cups-eq/d)	581	2.2 (0.12)	321	2.1 (0.14)
HEI-2015 score	581	52.4 (1.04)	321	50.4 (1.13)
Age (years)	581	28.6 (0.43)	321	28.7 (0.45)
Marital status (%)	580		321	
Married		69.7 (3.01)		67.4 (3.87)
Widowed/divorced/separated		1.8 (0.55)		4.6 (1.36)
Never married		28.5 (3.05)		28.1 (3.63)
Parity, number of live deliveries (%)	560		296	
0		26 (3.43)		23.4 (3.83)
1		40.9 (3.61)		37.7 (4.06)
≥ 2		33.1 (3.33)		39 (4.25)
Trimester (%)	581		271	
1st		26.5 (3.14)		42.8 (3.95)
2nd		33.6 (3.62)		26.2 (4.02)
3rd		39.9 (3.77)		31 (3.71)
Race/ethnicity (%)	581		321	
Mexican American/other Hispanic		21.9 (2.92)		19.4 (2.79)
Non-Hispanic White		55 (4.11)		56.1 (4.25)
Non-Hispanic Black		14.4 (2.71)		14.7 (2.64)
Other race (including multi-racial)		8.8 (2)		9.8 (2.1)
Education status (%)	581		321	
< High school		20.1 (2.27)		17.9 (2.5)
High school diploma		15.7 (1.79)		14.8 (2.93)
Some college		33 (3.31)		33.3 (3.95)
Undergraduate degree or higher		31.2 (3.41)		34 (4.21)
Household poverty income ratio (%)	551	62.7 (3.73)	306	63.7 (3.93)
Smoking (%)	581		321	
Never smoked		68.7 (3.34)		67.1 (4.51)
Past smoker		22.8 (3.36)		24.3 (4.26)
Current smoker		8.4 (1.65)		8.7 (2.17)
Physical activity (%)	578		318	
< 10 min/week		31.8 (3.54)		33.7 (4.13)
10 to < 150 min/week		29.2 (3.56)		28.9 (3.45)
≥ 150 min/week		38.9 (3.98)		37.5 (4.69)
Pre-pregnancy BMI (%)	581		285	
Underweight/normal		53.0 (3.1)		53.8 (4.2)
Overweight		20.9 (2.5)		22.6 (3.8)
Obese		26.1 (3.3)		23.6 (4.0)
Use of vitamin/mineral supplements (%)	581	84.3 (2.05)	321	76.5 (3.16)
History/diagnosis of diabetes (%)	581	1.6 (0.66)	0	0
Total energy (kcal)	581	2252 (54.9)	321	2223 (70.5)
Total fat (g)	581	83.5 (2.67)	321	85.9 (3.55)
Total fiber (g)	581	18 (0.72)	321	17.3 (0.71)
Total protein (g)	581	82.9 (2.14)	321	82 (2.48)
Added sugars (tsp.)	581	20.5 (0.96)	321	20.6 (1.23)
Gestational weight gain (N)	581		–	
Inadequate	114		–	
Adequate	178		–	
Excessive	289		–	
Gestational diabetes mellitus (N)	–		321	
Yes	–		57	
No	–		264	

Parentheses display standard errors

No statistically significant linear associations or linear trends between dairy consumption or HEI-2015 scores and either the odds of GDM or the prevalence of inadequate or excessive GWG were observed (Tables 2 and 3). When comparing women in Tertile 2 versus Tertile 1 of HEI-2015 scores, an increased prevalence of excessive GWG was observed in both Models 1 and 2 (Table 3).

**Table 2:** Adjusted odds ratios (and 95% confidence intervals) for diagnosis of gestational diabetes mellitus according to levels of total dairy consumption and HEI-2015 scores derived from multivariate logistic regression models adjusted for lifestyle, socioeconomic, and dietary confounders

Total dairy consumption	< 1 cup-eq/d	1 to < 2.5 cup-eq/d	≥ 2.5 cup-eq/d	P_Trend_
Model 1	1.0	1.22 (0.47, 3.13)	0.39 (0.11, 1.37)	0.132
Model 2	1.0	4.44 (0.94, 21.04)	0.64 (0.10, 4.04)	0.354
Model 3	1.0	4.30 (0.82, 22.5)	0.92 (0.10, 8.44)	0.764

HEI-2015 score [mean (SE)]	Tertile 1 36.9 (0.89)	Tertile 2 50.6 (0.39)	Tertile 3 66.0 (1.37)	P_Trend_
Model 1	1.0	1.52 (0.64, 3.62)	1.06 (0.41, 2.77)	0.872
Model 2	1.0	1.33 (0.42, 4.26)	0.88 (0.27, 2.87)	0.878

Model 1 is adjusted for age (and total energy for dairy consumption) (N = 321)

Model 2 is further adjusted for race/ethnicity, marital status, parity, education, poverty-income ratio, smoking status, physical activity, pre-pregnancy BMI, and vitamin/supplement use (N = 251)

Model 3 is further adjusted for total fat, fiber, protein, and added sugars (dairy consumption only) (N = 251)

Trend analyses were performed by imputing the median value of dairy consumption or HEI-2015 within each category

*cup-eq/d* cup-equivalents per day, *SE* standard error

**Table 3:** Adjusted prevalence ratios (and 95% confidence intervals) for classification of gestational weight gain according to levels of total dairy consumption and HEI-2015 scores derived from multivariate multinomial logistic regression models adjusted for lifestyle, socioeconomic, and dietary confounders

Total dairy consumption	< 1 cup-eq/d	Inadequate vs. adequate			Excessive vs. adequate		
		1 to < 2.5 cup-eq/d	≥ 2.5 cup-eq/d	P_Trend_	1 to < 2.5 cup-eq/d	≥ 2.5 cup-eq/d	P_Trend_
Model 1	1.0	1.10 (0.36, 3.32)	1.40 (0.44, 4.45)	0.562	1.90 (0.88, 4.07)	2.47 (0.88, 6.96)	0.129
Model 2	1.0	1.34 (0.41, 4.37)	1.24 (0.40, 3.83)	0.774	1.86 (0.77, 4.48)	2.16 (0.93, 5.01)	0.137
Model 3	1.0	1.62 (0.45, 5.86)	1.13 (0.32, 4.08)	0.994	1.98 (0.81, 4.84)	1.89 (0.79, 4.54)	0.301

HEI-2015 score [mean (SE)]	Tertile 1 38.6 (0.83)	Inadequate vs. adequate			Excessive vs. adequate		
		Tertile 2 50.5 (0.34)	Tertile 3 67.4 (0.88)	P_Trend_	Tertile 2 50.5 (0.34)	Tertile 3 67.4 (0.88)	P_Trend_
Model 1	1.0	1.30 (0.44, 3.86)	0.49 (0.17, 1.41)	0.179	3.36 (1.44, 7.85)	0.96 (0.43, 2.13)	0.760
Model 2	1.0	1.13 (0.36, 3.56)	0.36 (0.11, 1.22)	0.081	2.61 (1.10, 6.2)	0.96 (0.42, 2.19)	0.769

Model 1 is adjusted for age (and total energy for dairy consumption) (N = 581)

Model 2 is further adjusted for race/ethnicity, marital status, parity, education, poverty-income ratio, smoking status, physical activity, pre-pregnancy BMI, diagnosis/history of diabetes, and vitamin/supplement use (N = 530)

Model 3 is further adjusted for total fat, fiber, protein, and added sugars (dairy consumption only) (N = 530)

Trend analyses were performed by imputing the median value of dairy consumption or HEI-2015 within each category

*cup-eq/d* cup-equivalents per day, *SE* standard error

## Discussion

This study quantified the associations between consumption of dairy products and diet quality as measured by adherence to the 2015–2020 DGA with the odds of GDM and the prevalence of inadequate or excessive GWG among pregnant US women using nationally representative cross-sectional data. Despite the important role of diet in gestational health, intake of the nutrient-dense dairy component or overall diet quality were not associated with gestational outcomes.

The lack of associations between dairy consumption or diet quality and GDM is consistent with prior studies reporting null findings (Bao et al., 2013; Radesky et al., 2008; Schoenaker et al., 2016). In contrast, increased odds of GDM among women with a dietary pattern that included low milk and cheese intakes (Shin et al., 2015) and a decreased risk of GDM with increasing low-fat dairy intake (Osorio-Yáñez et al., 2017) have been reported.

No linear association between either dairy consumption or diet quality and GWG was observed. Previous reports with null associations between diet quality and GWG have relied upon an older HEI (Shin et al., 2014) or were conducted in a limited cohort that was not generalizable to the US population (Schlaff et al., 2020). Excessive GWG has been associated with higher energy intake and specific macronutrient subgroups (i.e., higher-carbohydrate, lower-fat intakes, and diets richer in fruits) (Lai et al., 2019; Wei et al., 2019), and with a higher HEI score modified to reflect the Malaysian diet in a non-US population (Yong et al., 2019). In the current study, however, no association was observed between excessive GWG prevalence and women with the highest HEI-2015 scores (relative to women with the lowest scores). The statistically significant association observed for excessive GWG among women with moderate (Tertile 2) HEI-2015 scores was unexpected. Different groups of individuals can all have HEI-2015 scores in the middle of the range, while individuals with lower or higher scores are more likely to be a homogenous group (Reedy et al., 2018); therefore, this finding should be interpreted with caution. When focusing on the dairy component, no association was observed. Previous studies have identified important interactions between pre-pregnancy BMI and dietary patterns for GWG (Liu et al., 2016; Parker et al., 2019), which could also explain the observation with HEI-2015. While statistically significant positive associations were noted between both inadequate and excessive GWG and pre-pregnancy obesity (relative to underweight/normal weight) in the adjusted models (data not shown), the limited sample size precluded multivariate analyses stratified by pre-pregnancy BMI. GWG could not be classified in the most recent NHANES (gestational age was not captured in cycles 2013–2016) and thus the impact of revised dietary recommendations as reflected in the HEI-2015 could not be fully assessed.

The lack of associations and inconsistencies with previous findings may be due to several factors including exposure and outcome misclassification, self-report of pre-pregnancy bodyweight and dietary intake, residual confounding, and sample size constraints; the last being a particular concern for the GDM analyses. Reliance on day 1 dietary recalls may result in misclassification of women into dairy and HEI-2015 categories by failing to identify all consumers of dairy or to accurately capture usual dietary patterns. Differential reporting bias (*e.g.*, pregnant women with relatively unhealthy eating behaviors may be more likely to exaggerate their diet quality) may have also impacted the findings, including the non-linear association between GWG and HEI-2015. Investigations into whether reporting bias of dietary components is unique among pregnant women are limited (Nowicki et al., 2011). The mixture of all dairy types with varying nutrient composition may contribute to the null findings if differences in the relationships within dairy types exist. Similarly, investigation of each HEI-2015 component score could provide more insight into the observed association with diet quality (Reedy et al., 2018). The classification of GDM and GWG in the current analysis, while based on national guidance, differs from other studies which may impact the findings and interpretation of results. GDM diagnosis in this analysis is based only on FPG as no OGTT was performed. Additional OGTT measurements can also be used to diagnose GDM. Therefore, we may have undercounted the number of GDM cases. Given that this misclassification occurred independent of the dietary recall, our results are likely biased towards the null. Finally, due to the cross-sectional design of NHANES, all measurements used to define the exposure and outcome variables in the current analysis are collected at one point in time during a woman’s pregnancy and assumes her diet does not change throughout.

In conclusion, the current investigation of the associations between dairy consumption or the HEI-2015 and the gestational health outcomes of GDM and GWG resulted in overall null findings. Given the limitations associated with the observational data used in the analyses, future research should be explicitly designed to minimize the misclassification and reporting bias that may have impacted the observed associations. The relationship between diet quality, distinct dietary components, and gestational health requires further investigation with data collection methods designed to measure the key exposure and outcome variables throughout pregnancy.

## Data Availability

The NHANES dietary recall, examination, interview, and laboratory data described in the article and used in the analysis are publicly available from the CDC via: https://wwwn.cdc.gov/nchs/nhanes/ContinuousNhanes/Default.aspx.

## Code Availability

STATA code is available upon request.
